# A Surface Hydrothermal Source of Nitriles and Isonitriles

**DOI:** 10.3390/life14040498

**Published:** 2024-04-11

**Authors:** Paul B. Rimmer, Oliver Shorttle

**Affiliations:** 1 Cavendish Laboratory, University of Cambridge, JJ Thomson Ave, Cambridge CB3 0HE, UK; 2 Institute of Astronomy, University of Cambridge, Cambridge CB3 0HA, UK; 3 Department of Earth Sciences, University of Cambridge, Cambridge CB2 3EQ, UK

**Keywords:** origin of life, volcanism on the early Earth, hydrothermal vents, graphite

## Abstract

Giant impacts can generate transient hydrogen-rich atmospheres, reducing atmospheric carbon. The reduced carbon will form hazes that rain out onto the surface and can become incorporated into the crust. Once heated, a large fraction of the carbon is converted into graphite. The result is that local regions of the Hadean crust were plausibly saturated with graphite. We explore the consequences of such a crust for a prebiotic surface hydrothermal vent scenario. We model a surface vent fed by nitrogen-rich volcanic gas from high-temperature magmas passing through graphite-saturated crust. We consider this occurring at pressures of 1–1000bar and temperatures of 1500–$1700\;{\;}^{\circ }C$. The equilibrium with graphite purifies the leftover gas, resulting in substantial quantities of nitriles (0.1% HCN and 1ppm HC_3_N) and isonitriles (0.01% HNC) relevant for prebiotic chemistry. We use these results to predict gas-phase concentrations of methyl isocyanide of ∼1 ppm. Methyl isocyanide can participate in the non-enzymatic activation and ligation of the monomeric building blocks of life, and surface or shallow hydrothermal environments provide its only known equilibrium geochemical source.

## 1. Introduction

Because the synthetic chemistry at life’s origins is a many-step process, a requirement for a prebiotic environment is clean, productive chemistry. If the prebiotic environment is too diverse and complex (if the number of different reacting species is too large), then the chemical parameter space inhabited by a geochemical environment becomes large, and the desired products and intermediates are lost in a morass of many thousands of other molecules. This is what we mean by messy chemistry: chemistry of such diversity and complexity that desired chemical products and behaviors are hard to realize [1,2,3].

The requirement for clean chemistry is related to the arithmetic demon: if a step-wise reaction does not provide products with high and selective yields and does not have a way of purifying and preserving desired products, then as the fraction of useful product becomes the reactant for the next step, subsequent yields become exponentially diluted. Chemical reaction yields of 10% over a sequence of twenty steps will take a starting solution with reactants at high concentration (1M) to a final product with a concentration of less than a molecule per cubic centimeter of solution. At some point, this ceases to be chemistry and becomes homeopathy.

Prebiotic chemistry is more likely to be successful when it is clean and productive. This places constraints on the environment in which successful prebiotic chemistry can occur. A prebiotic environment that hosts clean chemistry is one that facilitates the occurrence of selective, high-yield chemical reactions. A productive environment for prebiotic chemistry is one that facilitates the synthesis of complex organic molecules. These two environmental conditions are in tension with each other. The only conditions that have been experimentally demonstrated to be productive are reducing environments, and reducing environments tend to be messy. We will keep this tension in mind as we consider one of the particularly promising chemical starting points for prebiotic synthesis: nitriles.

Nitriles feature prominently in the chemistry of the majority of prebiotic systems [4,5]. They carry the same redox state as the nitrogen found in biomolecules [6] and have remained a constant in the highly fruitful iterative discovery of geochemically plausible UV-driven prebiotic synthesis of life’s monomeric building blocks [6,7]. Isonitrile chemistry, specifically chemistry involving methyl isocyanide, has been discovered to have the astounding chemical properties of activating and ligating monomeric building blocks [8], including nucleotides [9], phospholipids [10], and amino acids [9,11].

Paradoxes often bear fruit in scientific exploration [12]. In addition to clean, productive chemistry, ideal conditions for the prebiotic chemistry that forms nitriles admit several other paradoxes.

- Access to ultraviolet light at wavelengths between 200–400nm [13] and
  shielding from ultraviolet light at wavelengths between 200–400nm [8].
- Near-freezing temperatures [3,14,15,16] and
  near-boiling temperatures [17].
- Low pH [18] and
  neutral-to-high pH [18].
- Water activity∼1 as the conditions under which most nitrile-based prebiotic chemistry takes place and
  water activity ≪1 required for the phosphorylation of nucleosides and helpful for other condensation reactions [19].

It is not possible for a single static environment to fulfill all of these conditions. These can be satisfied, in principle, by a dynamic and heterogeneous environment. We show that surface hydrothermal vents fed by gas from high-temperature magma on the early Earth can qualify.

Surface hydrothermal vents are exposed to ultraviolet light where they are in contact with the atmosphere and shielded from ultraviolet light at depth or in crevices. Hydrothermal systems like the glaciovolcanic hydrothermal vents in Iceland today have a wide range of temperatures, pH values, and chemistries and can provide good analogues for anoxic systems from the past [20,21]. For a single hydrothermal vent, temperatures can be near freezing at its surface and above boiling at greater depths. Fluid flow through small channels of rock with natural mineral buffers towards a surface in contact with CO_2_ or phosphate-rich alkaline lake water [22] can provide a steep gradient from high to low pH. Most of the vent has water activity near unity, but the surface edges of the vent can dry or freeze, lowering the water activity.

Hydrothermal vents, whether shallow surface vents or underwater vents, have redox gradients generated by serpentinization and radiolysis [23,24]. It is unlikely that these processes would have generated sufficiently reducing conditions for the generation of nitriles or isonitriles [23,25,26]. Primordial abiotic kerogen could provide sufficient reducing power, but it is debatable whether significant amounts of kerogen was present in the crust or upper mantle before life [27,28].

Even if reducing conditions are accessible, it is unlikely that they would result in selective chemistry [26]. Often, the choice is reducing conditions or chemical selection. Clean, selective chemistry tends to be oxidizing, or at least neutral. Reducing chemistry often results in tar [29].

In this paper, we show that clean, productive chemistry rich in nitriles and isonitriles can be found together in surface hydrothermal vents because of graphitization. We present the surface vent scenario in Section 2. We discuss the model used to predict the surface vent chemistry in Section 3 and show our results in Section 4. Section 5 contains a discussion and conclusions.

## 2. The Scenario

**Figure 1:**
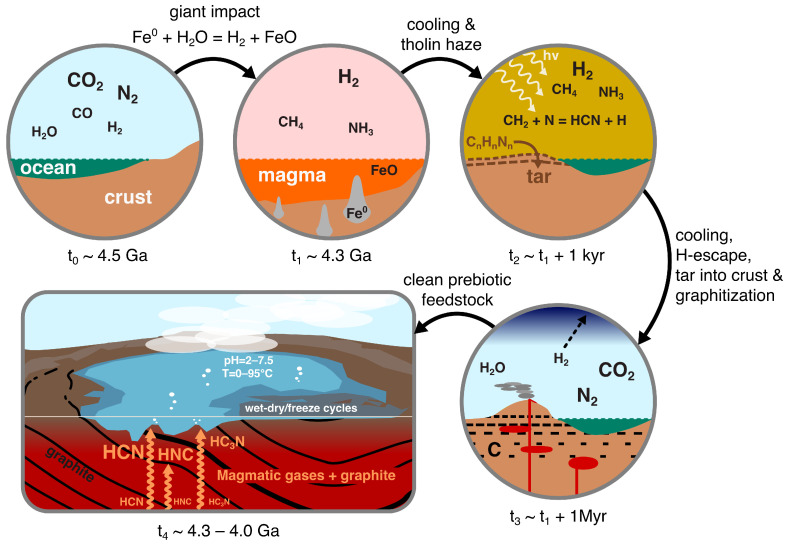
A schematic representation of the scenario we propose here for clean, high-yield production of prebiotic feedstock. Events move around clockwise from the top left: First, the Earth has a neutral atmosphere. This is reduced following a giant impact at 4.3 Ga by oxidation of the impactor’s metal core to produce a massive H_2_ atmosphere with significant methane and ammonia. This atmosphere quickly cools (in <1 kyr), with photochemistry producing a tholin-rich haze that deposits complex nitrogen-rich organics. These organics become progressively buried and graphitized by interaction with magma. The atmosphere clears as H_2_ is lost to space and becomes neutral again. Finally, magmatic gases interact with the graphite and are scrubbed to produce high yields of clean HCN, HC_3_N, and isonitriles.

We present a scenario that we predict to result in clean and productive prebiotic chemistry. It is a scenario that is both prebiotically plausible and well supported by observations, experiments, and models. A schematic of this scenario is given in Figure 1. We will discuss some of the simplifying assumptions of this scenario and other ways the same chemistry could emerge in Section 5.

The Hadean Eon spans 500 million years of Earth’s history: from planet formation to 4 Ga. After the moon-forming impact at ∼4.5 Ga, Earth likely had an atmosphere dominated by CO_2_ and N_2_, with some CO and H_2_O [30], around 1–5% H_2_ [31], and comparatively trace amounts of sulfur-bearing compounds SO_2_ and H_2_S [32], with low-to-mid, stable concentrations of sulfites in most natural waters [33].

At a time around ∼4.3 Ga, Earth was likely hit by a roughly moon-sized object [34]. The iron in this giant impactor would have reacted with ocean water, producing large amounts of hydrogen at high temperatures. Such a hydrogen-dominated atmosphere equilibrates with the surface magma generated to give ∼1 bar partial pressure of H_2_ [35]. In the high temperatures of this post-impact atmosphere, hydrogen would have reacted with carbon dioxide and nitrogen to produce methane and ammonia [36,37]. These giant impacts thereby initiated transient and global highly reducing conditions in the atmosphere and on the crust of the Hadean Earth. Many of the reducing molecules, hydrogen, methane, and ammonia are greenhouse gases, and the surface of Earth at this time would have been hot: likely above the boiling point of liquid water at 1 bar pressure [36].

In these conditions, the hydrogen, methane, and either nitrogen or ammonia in the atmosphere would have been photodissociated, with their products combining to produce the nitriles HCN and HC_3_N [37]. Many complex organics would also have formed during this epoch; these would condense out of the atmosphere, forming a tholin-like haze [36,38,39]. This nitrogen-rich haze would have rained out onto the hot surface as a thick tar [36,40,41]. It is likely some of this tar was incorporated into the crust, either by tectonic, magmatic, or impact churning of the surface. Over the period of a million years or more, the atmosphere would have returned to a neutral chemical state through the conversion of methane and ammonia back to carbon dioxide, nitrogen, and hydrogen and due to the escape of hydrogen into space [36,37]. The surface of Earth would then cool to near freezing [42].

Photochemically produced tar mixed into the crust would have experienced episodic heating to >1500 ${\;}^{\circ }$C by the early high-temperature (komatiitic) magmas known to have been an important constituent of early Earth magmatism [43]. We will show that this heating likely broke apart the tar, transforming most of it into graphite, molecular hydrogen, and molecular nitrogen (though some of the hydrogen and nitrogen may have been complexed with the graphite at this stage). Magmatic gas, interacting with the graphitized crustal material, was likely transformed into HCN, HC_3_N, and isonitriles along with sulfide and carbon monoxide and little else. The result is clean, productive chemistry, which could degas through shallow and surface hydrothermal vents on ancient volcanic islands.

## 3. The Model

We will now model the latter part of this scenario: the interaction of gas initially in equilibrium with $f_{O_{2}}\approx \mathrm{QFM}-1$ magma (i.e., one log unit below the quartz–magnetite–fayalite buffer in terms of its oxygen fugacity) as it flows through graphite. We start with a fiducial model at $1700\;{\;}^{\circ }C$, 100 bar gas at QFM−1, plus a fixed nitrogen content of $\begin{array}{c} N \end{array}=5.7\%$. Graphite is added to the system. QFM−1 conditions are incorporated by setting initial conditions to the values from Table 1.

We are not here invoking nitrogen-rich magma. The nitrogen is included in the magmatic gas only for convenience; it makes no difference if it is included initially or included with the graphite because the quantity is not varied. The source of the nitrogen is expected to be the same as the source of the graphite. This nitrogen concentration represents the predicted nitrogen-rich nature of the post-impact organics [37] that were then incorporated into the crust.

To predict the changes in the concentrations of chemical species as a function of added graphite, we solve the following equation:

(1) $$\frac{d[X]}{dt}=P_{X}-L_{X}\left[X\right],$$

where $\left[\begin{array}{c} X \end{array}\right]\;\left({\mathrm{cm}}^{-3}\right)$ is the concentration of species X, $P_{X}\;\left({\mathrm{cm}}^{-3}\;s^{-1}\right)$ is the rate of production for that species, and $L_{X}\;\left(s^{-1}\right)$ is the rate of loss for that species. The terms $P_{X}$ and $L_{X}$ are made up of concentrations of other species and rate constants *k*, which themselves have units depending on the reaction order and, when reversible, are set to reproduce chemical equilibrium by assuring that, for the generic reaction:

(2) A+B+C+…⇄X+Y+Z+…,

with forward rate constant $k_{+}$ and reverse rate constant $k_{-}$, the reverse rate constant is set such that:

(3) $$\frac{k_{+}}{k_{-}}=K_{\mathrm{eq}}=e^{-\Delta_{f}G/RT}=\frac{\{X\}\{Y\}\{Z\}\ldots }{\{A\}\{B\}\{C\}\ldots },$$

where $\left\{\begin{array}{c} A \end{array}\right\}$, etc., are the activities of the different chemical species, and $\Delta_{f}G/RT$ is determined using NASA coefficients, mostly from Burcat and Ruscic [44], and subsequent updates to the database. More details can be found in prior presentations of the underlying model [45,46].

For these calculations, we use an updated gas-phase chemical network based on STAND-2020 [46]. This model includes H/C/N/O/S chemistry, a very limited P network, and some reactions involving various heavier elements such as Fe, Mg, and Ti. It includes 6279 reactions involving 511 chemical species. The full list of species and the network are available at https://doi.org/10.7910/DVN/FKKYY3 (accessed on 28 March 2024). The main addition is the two reactions:

(4) C(g)⇄C(s),

where C(g) is gas-phase carbon and C(s) is solid carbon as graphite and the rate constants are set to reproduce equilibrium. The model is run for one day model time.

The kinetics model and the FastChem (v3.1) model use different vapor pressures for graphite. For the kinetics model, we use [47,48]:

(5) $$\log_{10}\;p_{\mathrm{vap}}=6.455-\frac{2.7709\times 10^{4}}{T-3.549}-\frac{10^{7}}{T^{2}},$$

where p(bar) is the pressure and T(K) is the temperature.

We want to compare our kinetics results to equilibrium. We predict the equilibrium of the magmatic gas using FastChem (v3.1) [49], to which we have added the thermochemical data of cyanoacetylene (HC_3_N) [50] given as the constant of mass action

(6) $$\ln K(T)=\frac{2.96\times 10^{5}}{T}-4.83\ln T-28.47+\left(1.91\times 10^{-3}\right)\;T-\left(1.10\times 10^{-7}\right)\;T^{2},$$

where *T* is the temperature in Kelvin. For FastChem (v3.1), the vapor pressure works out to be approximately:

(7) $$\log_{10}\;p_{\mathrm{vap},\mathrm{FC}}=4.855-\frac{2.5709\times 10^{4}}{T}-\frac{10^{7}}{T^{2}}.$$

We run our model for a wide range of conditions to determine the sensitivity of our results to elemental composition, temperature, and pressure. We run the model from $1300\;{\;}^{\circ }C$ to $1800\;{\;}^{\circ }C$ and from 1bar to 1000bar, varying H, C, N, and O elemental abundances.

Our network does not include methyl isocyanide (CH_3_NC), the prebiotically relevant compound, and the gas-phase kinetics of this species would require more investigation before they could be reliably included in this model. We show HNC as an indication of the overall concentration of isonitriles, and we use the BURCAT thermochemistry data to estimate the concentration of CH_3_NC.

We can use the Gibbs free energy $\left(\Delta_{r}G,\;\mathrm{kJ}/\mathrm{mol}\right)$ for the following reaction:

(8) $$HCN+CH_{4}\to CH_{3}NC+H_{2},$$

to predict the CH_3_NC concentration. This can be expressed as:

(9) $$\left[CH_{3}NC\right]=e^{-\Delta_{r}G/RT}\frac{\left[HCN][CH_{4}\right]}{\left[H_{2}\right]}.$$

where [HCN] and [CH_4_] are the concentrations of HCN and CH_4_, respectively; R=8.3145 J/ (mol K) is the gas constant, and T(K) is the temperature.

## 4. Results

**Figure 2:**
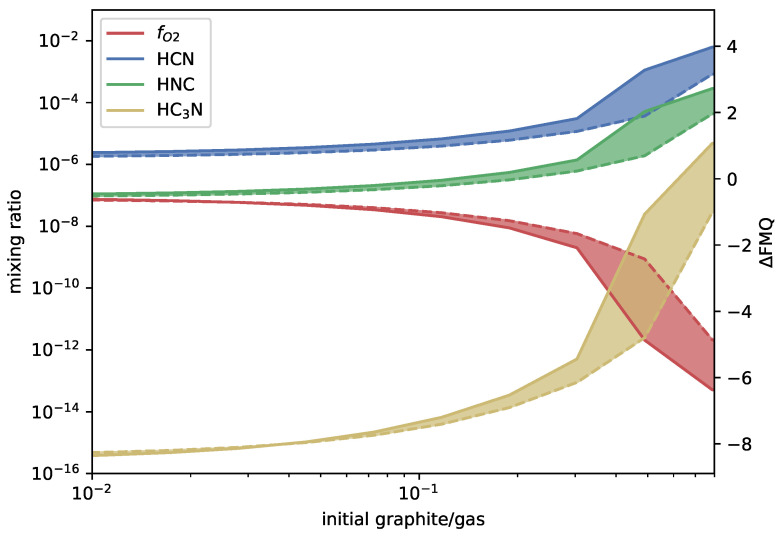
Mixing ratios of HCN, HNC, and HC_3_N (left y-axis) as a function of added graphite (x-axis) for a QFM-1 buffered magmatic gas with 5.7% elemental nitrogen content held at $1700\;{\;}^{\circ }C$ and 100bar. The right y-axis shows the deviation from QFM in log units. Solid lines indicate the kinetics (this paper) results and dashed lines the equilibrium (FastChem) results, with the range between the two shaded in.

**Figure 3:**
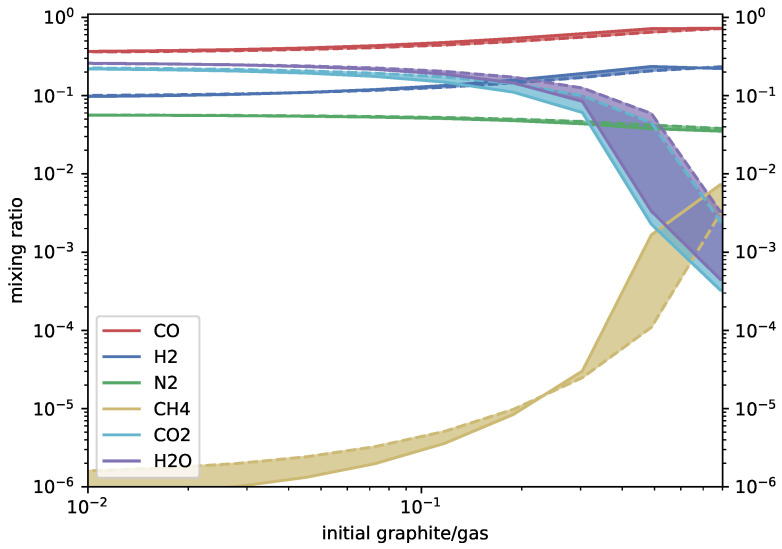
Mixing ratios of major species (y-axis) for a QFM-buffered magmatic gas with 5.7% elemental nitrogen content held at $1700\;{\;}^{\circ }C$ and 100bar. Solid lines indicate the kinetics (this paper) results and dashed lines the equilibrium (FastChem, v3.1) results, with the range between the two shaded in.

At $1700\;{\;}^{\circ }C$ and 100 bar, the model achieves equilibrium after $10^{3}$–$10^{4}\;s$ when the graphite concentration is ≲0.1. The kinetics and equilibrium models then diverge. The equilibrium model does not change with graphite added beyond a concentration of 0.8, which is the saturation limit of graphite. The kinetics model continues to deviate from equilibrium, but much more slowly. The results below saturation are given in Figure 2 for HCN, HNC, and HC_3_N and Figure 3 for the major species. A datafile of the full results is available at https://doi.org/10.7910/DVN/FKKYY3 (accessed on 28 March 2024). The results are given as a function of carbon and oxygen fractions.

**Figure 4:**
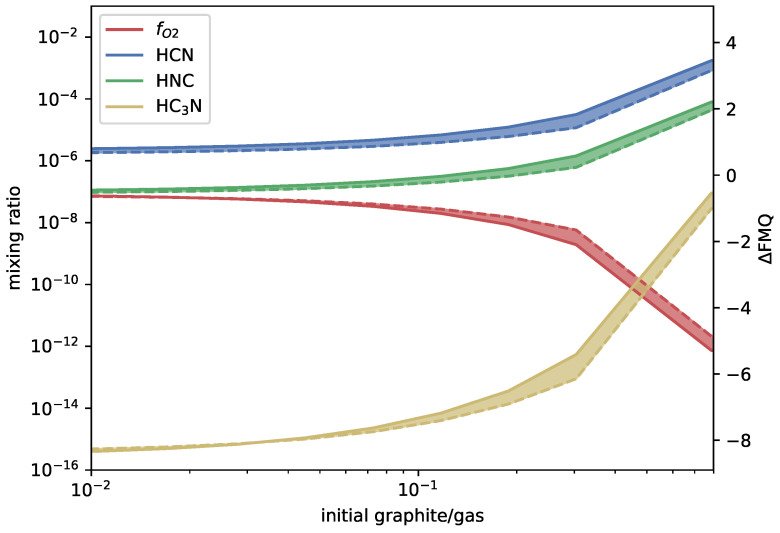
Mixing ratios of HCN, HNC, and HC_3_N (left y-axis) as a function of added graphite (x-axis) for a QFM-buffered magmatic gas with 5.7% elemental nitrogen content held at 1700 ${\;}^{\circ }$C and 100bar. Here, the kinetics model and the equilibrium model both use graphite vapor pressure equal to Equation (6). The right y-axis shows the deviation from QFM in log units. Solid lines indicate the kinetics (this paper) results and dashed lines the equilibrium (FastChem, v3.1) results, with the range between the two shaded in.

The main reason for the deviation between the two models is the choice of graphite vapor pressure, where the kinetics model uses Equation (5) and the equilibrium model uses Equation (6). If the kinetic model is run with the graphite vapor pressure equal to Equation (6), the results converge, as can be seen in Figure 4.

**Figure 5:**
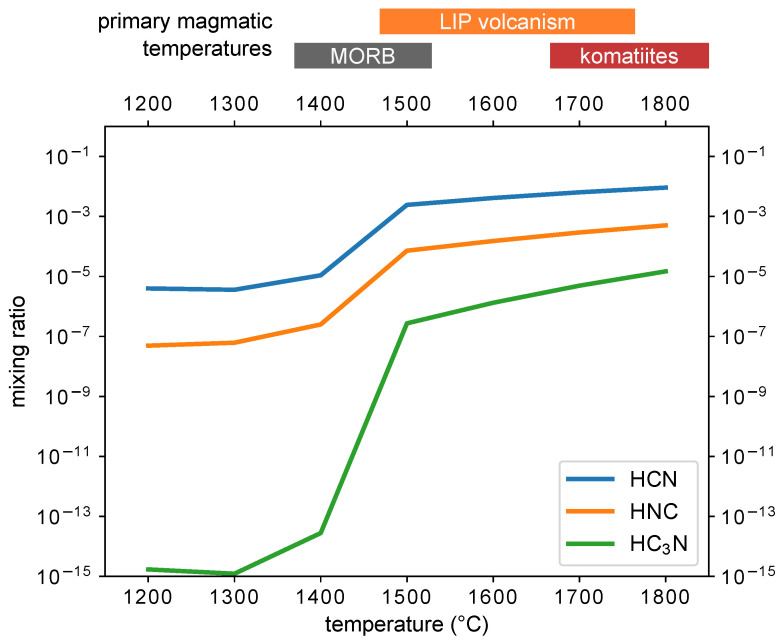
Mixing ratios of HCN, HNC, and HC_3_N (y-axis) at graphite saturation as a function of temperature (${\;}^{\circ }$C, x-axis) for a magmatic gas starting at QFM−1 with 5.7% elemental nitrogen content held at 100bar. Only the kinetics results are shown. Indicative eruptive temperatures of terrestrial magmas are given above the plot: ‘MORB’ = mid-ocean ridge basalts [51], ‘LIP’ = large igneous province [51], and komatiite temperatures are given for Archean-age examples [43].

We also show how the concentrations of HCN, HNC, and HC_3_N at graphite saturation depend on the magma temperature; see Figure 5. The results of the sensitivity analysis are presented in Appendix A. This analysis was only run for the chemical kinetics model.

The most important result is that graphite formation cleans up the chemistry considerably. While Rimmer and Shorttle [26] predict that the majority carbon-containing species is diacetylene, our study finds that the majority carbon-containing species is graphite, and diacetylene has decreased from 42% to <0.1%. This is significant because diacetylene at lower temperatures will polymerize and will plausibly generate a mass of large inert hydrocarbons: effective “tarrification” [29]. Graphite formation resolves much of this problem. Since graphite is removed from the gas phase, the remaining thermochemically stable gas is enriched, and so much higher concentrations of cyanide (HCN) and cyanoacetylene (HC_3_N) are achieved: to a maximum of ∼1% and ∼1 ppm of the gas-phase, respectively.

An unexpected prediction is the formation of higher-than-expected concentrations of hydrogen isocyanide (HNC). At graphite saturation, $\left[\begin{array}{c} HNC \end{array}\right]$∼0.01%.

**Figure 6:**
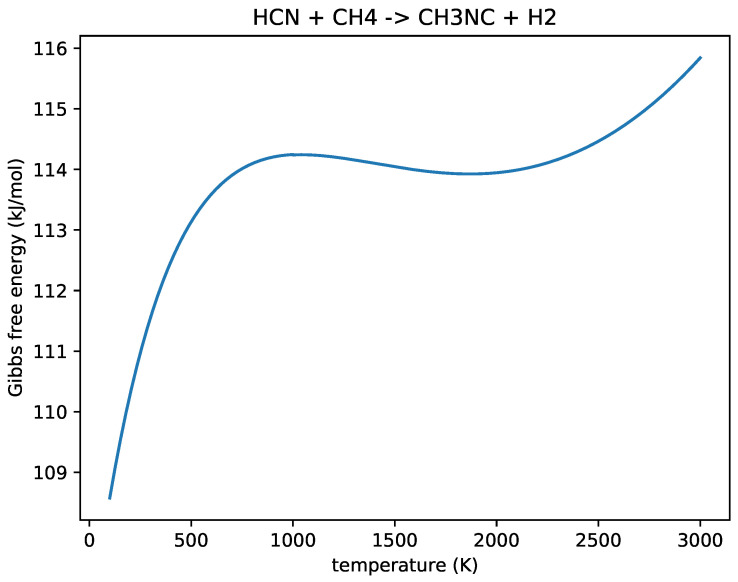
The Gibbs free energy of the reaction: $\begin{array}{c} HCN \end{array}+\begin{array}{c} CH_{4} \end{array}\to \begin{array}{c} CH_{3}NC \end{array}+\begin{array}{c} H_{2} \end{array}$ (kJ/mol) as a function of temperature (K). We can see that for all temperatures, this formation reaction for CH_3_NC is endergonic, but not strongly so.

We use the data from BURCAT to calculate the Gibbs free energy of Reaction (8) and show this free energy in Figure 6. We apply the value of the Gibbs free energy at $1700\;{\;}^{\circ }C$ of 114kJ/mol, and $[\begin{array}{c} H_{2} \end{array}]=0.2$, $[\begin{array}{c} CH_{4} \end{array}]=$ 3–7 $\times 10^{-3}$ and $[\begin{array}{c} HCN \end{array}]=$$10^{-3}$–$10^{-2}$ for Equation (9) to predict the equilibrium concentration: $\left[\begin{array}{c} CH_{3}NC \end{array}\right]$ is between $7\times 10^{-7}$ and $2\times 10^{-5}$.

At lower pressure, if the magma is graphite saturated, then HCN, HNC, and HC_3_N remain effectively unchanged. In systems that are not graphite saturated, HCN and, in the right range, HNC, become even more favored, with HNC achieving mixing ratios of ∼0.1% of the gas phase. Cyanoacetylene drops significantly—by roughly two orders of magnitude per order of magnitude of decreasing pressure—to ≲100 ppt levels at 1 bar. In any event, experiments to test the predictions of this model at ambient pressure when the system is graphite saturated may be tractable in the near future.

At lower temperatures, graphite reigns. Rimmer and Shorttle [26] predict that at 1200 ${\;}^{\circ }$C, significant amounts of HCN and HC_3_N could still be formed at >10 bar pressures. With our present model, the carbon is largely removed into graphite, and species like methane, molecular hydrogen, and carbon monoxide dominate. For model results over a full range of oxygen, carbon, and hydrogen fractions, see Appendix A.

## 5. Discussion and Conclusions

In this paper, we present a new calculation of magmatic gas-phase chemistry for nitrogen-rich but otherwise standard oxidation state (QFM−1) magmas at 1700 ${\;}^{\circ }$C temperatures and 100 bar pressure with graphite added to the point of saturation. This is the specific sequence we have modeled, but it may well turn out that the QFM−1 magma is nitrogen-poor, and instead, at temperatures of ∼1700 ${\;}^{\circ }$C, nitrogen is made available to the gas from the its complex with graphite. This model differs from Rimmer and Shorttle 2019 [26] in that we now include phase equilibrium with graphite. We find that graphite helps to clean up the chemistry and increases the concentrations of prebiotically relevant compounds, nitriles, and isonitriles if the magma temperature is ∼1700 ${\;}^{\circ }$C, consistent with early experimental studies of nitrogen and graphite at high temperatures [52]. If the magma temperature is lower, the concentration of nitriles and isonitriles drops significantly; see Figure 5.

Including graphite is not predicted to result in a decrease in nitriles so long as the temperature is sufficiently high (≳1500 ${\;}^{\circ }$C). The results at lower temperatures indeed show significant suppression of nitriles in line with previous model expectations for hydrogen solubility, such as those of Wogan et al. [53]. It is important to note that these environments are locally at least an order of magnitude more reducing than the minimum $f_{\begin{array}{c} O_{2} \end{array}}$ considered by Wogan et al. Even under these conditions, the roughly estimated kg gas/kg magma for CH_4_ at 100 bar and 1700 K would be ≲$10^{-4}$, in reasonable agreement with Wogan et al. If the magma was still present at the point of gas–graphite interaction, solubility experiments would need to be performed under these unusual conditions in order to determine the true fate of gas-phase nitriles and other hydrogen- and nitrogen-bearing species.

**Figure A5:**
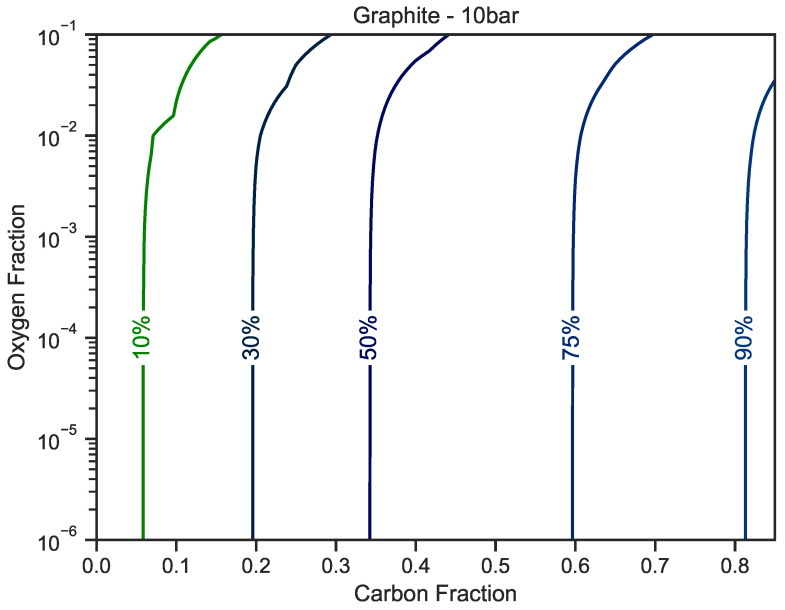
Fraction of graphite in the mixture as a function of carbon (x-axis) and oxygen (y-axis) fractions for a magmatic gas held at $1600\;{\;}^{\circ }C$ and 10bar. The nitrogen fraction is set to 5%. The hydrogen fraction makes up the difference, if any.

**Figure A6:**
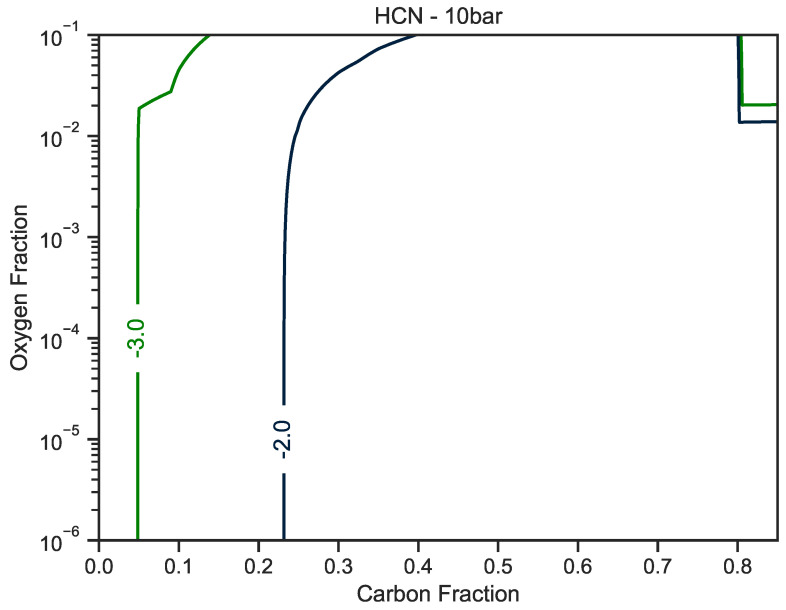
Hydrogen cyanide concentrations as $\log_{10}\left(\left[\begin{array}{c} HCN \end{array}\right]\right)$ as a function of carbon (x-axis) and oxygen (y-axis) fractions for a magmatic gas held at $1600\;{\;}^{\circ }C$ and 10bar. The nitrogen fraction is set to 5%. The hydrogen fraction makes up the difference, if any.

**Figure A7:**
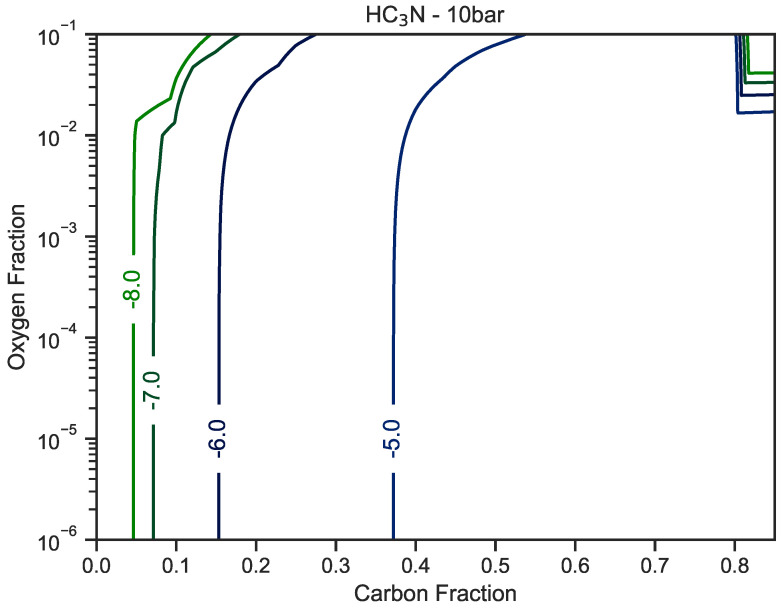
Cyanoacetylene concentrations as $\log_{10}\left(\left[\begin{array}{c} HC_{3}N \end{array}\right]\right)$ as a function of carbon (x-axis) and oxygen (y-axis) fractions for a magmatic gas held at $1600\;{\;}^{\circ }C$ and 10bar. The nitrogen fraction is set to 5%. The hydrogen fraction makes up the difference, if any.

**Figure A8:**
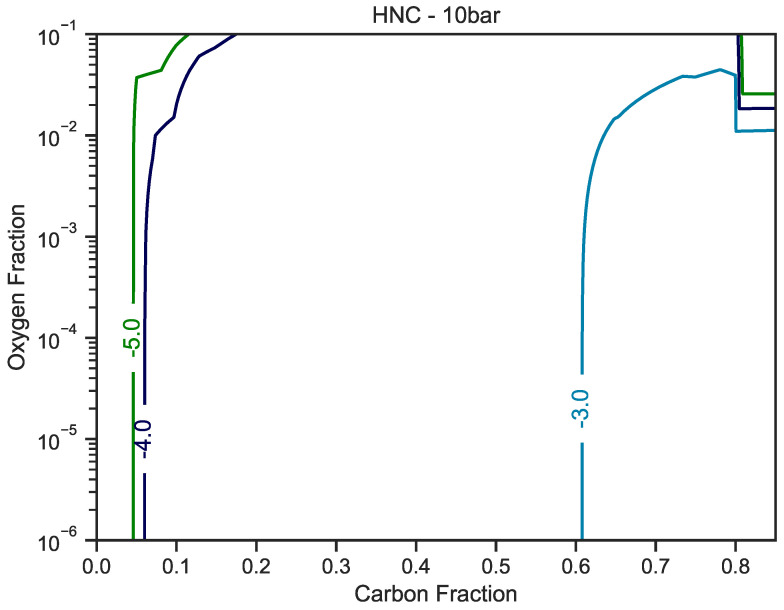
Hydrogen isocyanide concentrations as $\log_{10}\left(\left[\begin{array}{c} HNC \end{array}\right]\right)$ as a function of carbon (x-axis) and oxygen (y-axis) fractions for a magmatic gas held at $1600\;{\;}^{\circ }C$ and 10bar. The nitrogen fraction is set to 5%. The hydrogen fraction makes up the difference, if any.

**Figure A9:**
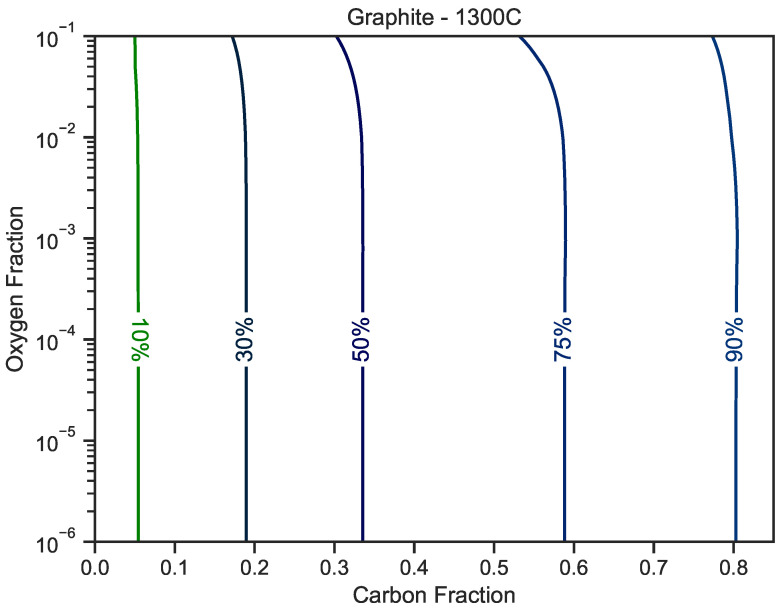
Fraction of graphite in the mixture as a function of carbon (x-axis) and oxygen (y-axis) fractions for a magmatic gas held at $1300\;{\;}^{\circ }C$ and 100bar. The nitrogen fraction is set to 5%. The hydrogen fraction makes up the difference, if any.

**Figure A10:**
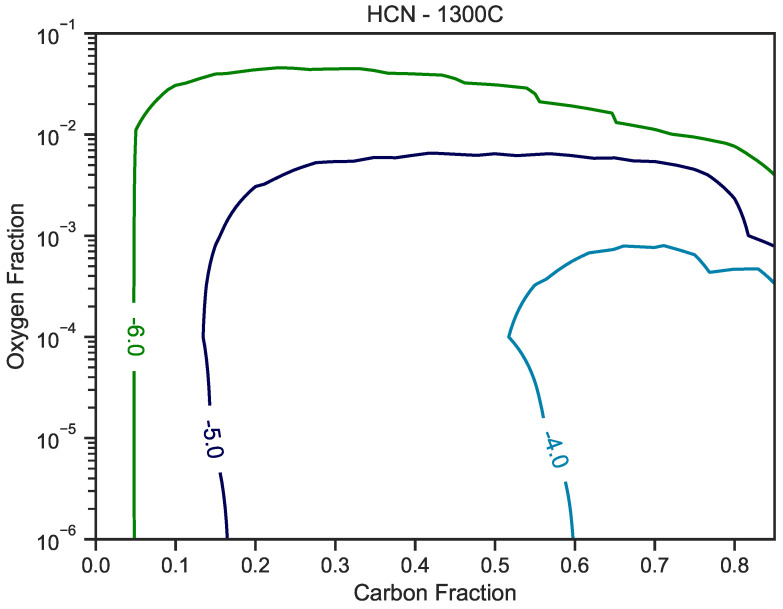
Hydrogen cyanide concentrations as $\log_{10}\left(\left[\begin{array}{c} HCN \end{array}\right]\right)$ as a function of carbon (x-axis) and oxygen (y-axis) fractions for a magmatic gas held at $1300\;{\;}^{\circ }C$ and 100bar. The nitrogen fraction is set to 5%. The hydrogen fraction makes up the difference, if any.

**Figure A11:**
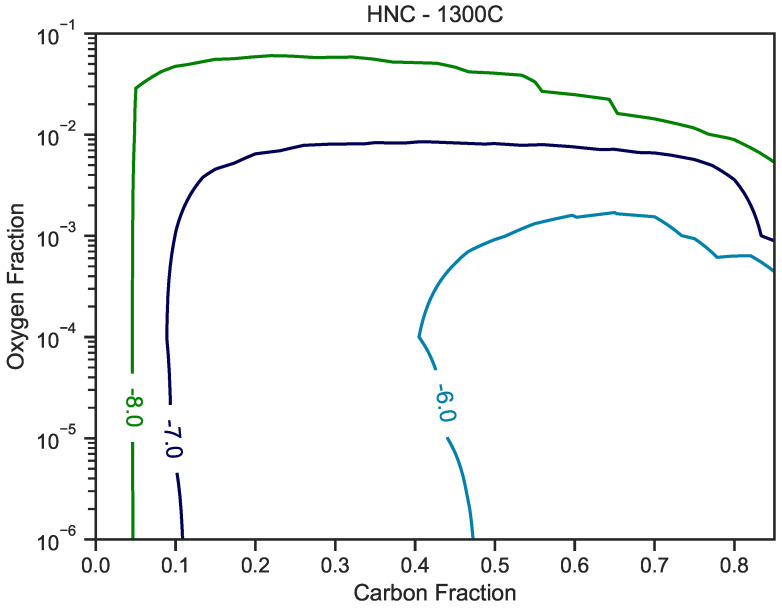
Hydrogen isocyanide concentrations as $\log_{10}\left(\left[\begin{array}{c} HNC \end{array}\right]\right)$ as a function of carbon (x-axis) and oxygen (y-axis) fractions for a magmatic gas held at $1300\;{\;}^{\circ }C$ and 100bar. The nitrogen fraction is set to 5%. The hydrogen fraction makes up the difference, if any.

The required conditions for clean chemistry with nitriles and isonitriles is graphite-saturated magma at ≳1500 ${\;}^{\circ }$C; otherwise, the carbon and nitrogen that would form nitriles are locked up in N_2_ and graphite. See the appendix for plots showing the dependence of the nitrile concentration on the temperature and concentration of carbon (Figure A9, Figure A10 and Figure A11). Cyanoacetylene is favored over a wide range of parameter spaces at higher pressures (≳100 bar—see Appendix A, Figure A5, Figure A6, Figure A7 and Figure A8)—but we do not know if we can predict that it can be produced in abundance at pressures as low as 1bar so long as the system is saturated with graphite. See also Table A1 for a summary of these comparisons.

The required high temperatures effectively limit the source of nitriles and isonitriles to more niche Hadean environments, particularly those that run at higher temperatures, such as Komatiite magmas [43,54], for which temperatures can surpass $1600{\;}^{\circ }$C [55]. These high-temperature magmas are thought to be more prevalent (though not ubiquitous) on the Hadean and Archaean Earth [56,57]. This development of the theory admits several potential scenarios beyond the one we presented in Section 2.

It may simply be that certain regions of the early upper mantle were highly reduced, possessing abiotically generated kerogen-like material, in a manner hypothesized by Thomas Gold, among others [27]. The natural high temperatures of early volcanic systems could heat and reprocess this material.

Cosmic dust is kerogen-like and would have been much more ubiquitous on early Earth, with as much as 50% of the dust being cosmic during the Hadean [58]. The chemistry predicted here could arise in environments where this dust is concentrated and then heated volcanically or by a subsequent large impact.

Radiolysis and serpentinization can generate large redox gradients [23,59], with certain regions of the crust becoming much more reduced while others become more oxidized. If the reduced regions intersected a magmatic flow at >10 bar, the heat would convert much of the chemistry to nitriles and isonitriles. Radiolysis especially would have been much more intense during the Hadean [60].

Though we favor the scenario presented in Section 2 for early Earth, based on current geological and experimental evidence, we are encouraged that similar prebiotic chemistry can emerge in a wide range of conditions expected on early Earth, Mars, and exoplanets. It is also worth noting that all of these scenarios are compatible with shallow hydrothermal systems, which admit most of the physical and chemical advantages of underwater hydrothermal events [61]. It is also worthy of note that some of these systems, such as the surface hydrothermal fields near the Erebus volcano, Antarctica, exhibit these vast temperature shifts, with high-temperature magmatic systems intersecting with vents that release gas into ice [21]: a system that would favor concentrating some of these prebiotic feedstocks into eutectic phases. In addition, these systems show fascinating, possibly abiotic redox behavior and can incorporate nitrogen from the atmosphere into their magmatic systems [21].

The provision of chemical feedstocks from high-temperature magmas may pave the way for order amidst geochemical chaos, with clean chemical equilibrium mixtures at high concentrations segregated by pools or streams on the basis of the magmatic source: magmas with higher carbon content provide isonitriles such as methyl isocyanide. All of this is mediated and regulated by the formation of graphite, which removes from the gas excess carbon and the combinatorial mess that comes with it, and high temperatures, which favor relatively simple gas-phase mixtures of the starting material required for productive prebiotic chemistry. Natural prebiotic environments need not produce messy chemistry; the environment can constrain the chemistry to be clean and productive. Even if the environments favoring more chemically ordered and promising prebiotic chemistry turn out to be a very small fraction of the total environment, the selection pressure for productive synthesis could outweigh their relative rarity.

Whether this chemical solution provides a “buffet lunch” for prebiotic chemistry or an unappetizing and unusable mess depends on the kinetic stability and solubility of the gas-phase mixture once it is quenched and enters into the surface waters. This question can only be resolved with future experiments.

## Figures and Tables

**Table 1:** Initial conditions for the kinetics model. We set temperature equal to $1700\;{\;}^{\circ }C$ and pressure is 100bar. The mixing ratios of species are given below. Abundances have been rounded. The exact abundances used can be found at https://doi.org/10.7910/DVN/FKKYY3 (accessed on 28 March 2024).

Species	C(g)	CO	H_2_	N_2_	O_2_	CH_4_	CO_2_	H_2_O
Mixing Ratio	*	0.35	0.1	0.057	$7.6\times 10^{-10}$	$1.4\times 10^{-6}$	0.23	0.26

* Graphite content is varied from zero to saturation (elemental abundance ≈ 80%)

**Figure A1:**
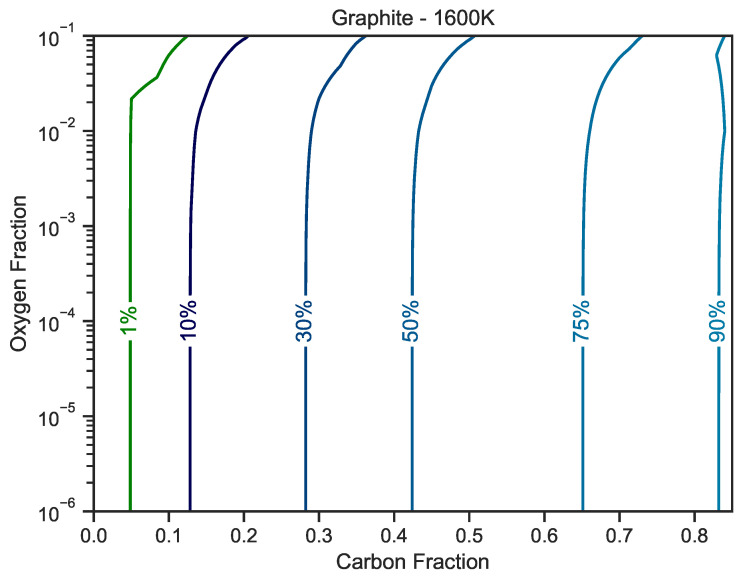
Fraction of graphite in the mixture as a function of carbon (x-axis) and oxygen (y-axis) fractions for a magmatic gas held at $1600\;{\;}^{\circ }C$ and 100bar. The nitrogen fraction is set to 5%. The hydrogen fraction makes up the difference, if any.

**Figure A2:**
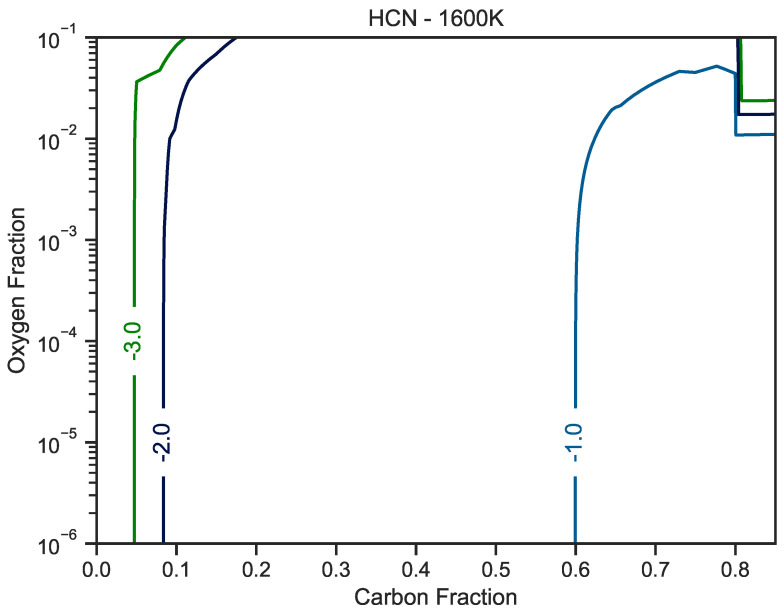
Hydrogen cyanide concentrations as $\log_{10}\left(\left[\begin{array}{c} HCN \end{array}\right]\right)$ as a function of carbon (x-axis) and oxygen (y-axis) fractions for a magmatic gas held at $1600\;{\;}^{\circ }C$ and 100bar. The nitrogen fraction is set to 5%. The hydrogen fraction makes up the difference, if any.

**Figure A3:**
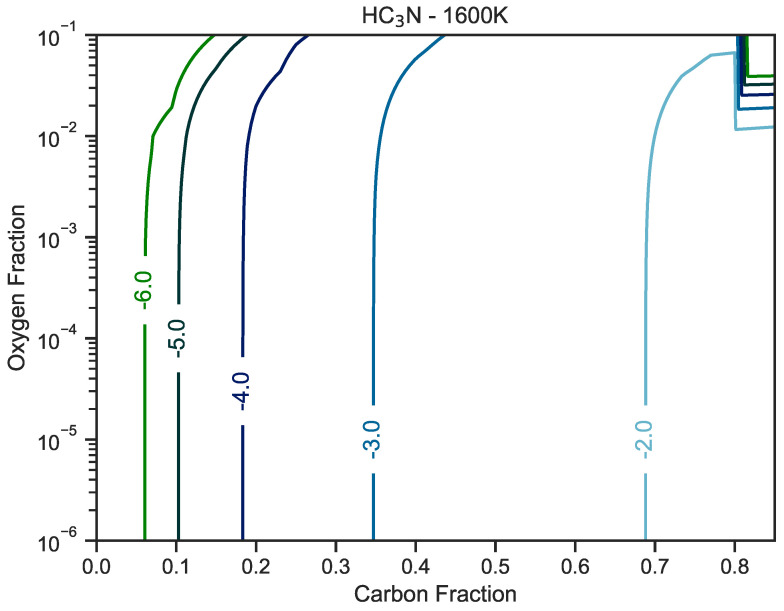
Cyanoacetylene concentrations as $\log_{10}\left(\left[\begin{array}{c} HC_{3}N \end{array}\right]\right)$ as a function of carbon (x-axis) and oxygen (y-axis) fractions for a magmatic gas held at $1600\;{\;}^{\circ }C$ and 100bar. The nitrogen fraction is set to 5%. The hydrogen fraction makes up the difference, if any.

**Figure A4:**
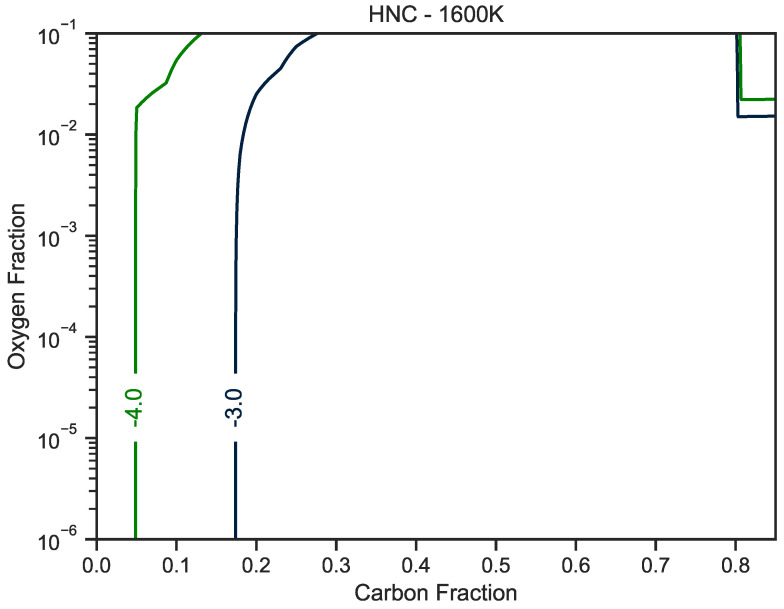
Hydrogen isocyanide concentrations as $\log_{10}\left(\left[\begin{array}{c} HNC \end{array}\right]\right)$ as a function of carbon (x-axis) and oxygen (y-axis) fractions for a magmatic gas held at $1600\;{\;}^{\circ }C$ and 100bar. The nitrogen fraction is set to 5%. The hydrogen fraction makes up the difference, if any.

## Data Availability

All data required to reproduce the results of this paper can be found at https://doi.org/10.7910/DVN/FKKYY3 (accessed on 28 March 2024). FastChem v3.1 can be found at https://github.com/exoclime/FastChem (accessed on 28 March 2024).

## Appendix A. Sensitivity Analysis

Here, we show the results of varying the gas-phase chemistry of the magma by adjusting the elemental abundances of H, C, and O with a fixed N at 5%. These results are shown for a pressure of 100 bar and a temperature of $1600{\;}^{\circ }C$ (Figure A1, Figure A2, Figure A3 and Figure A4) as well as for a pressure of 10 bar and a temperature of $1600{\;}^{\circ }C$ (Figure A5, Figure A6, Figure A7 and Figure A8) and for a pressure of 100 bar and a temperature of $1300{\;}^{\circ }C$ (Figure A9, Figure A10 and Figure A11). For the last case, HC_3_N is not shown because its abundance is below 1 ppm over the entire parameter space. Sensitivities based on these results are summarized in Table A1. The full results including all molecules predicted by the model and network are given at https://doi.org/10.7910/DVN/FKKYY3 (accessed on 28 March 2024).

**Table A1:** Sensitivity of species concentrations on temperature and pressure, expressed as ratios. [X*_a_*/X*_b_*] is the ratio of $\left[\begin{array}{c} X \end{array}\right]$ under the conditions specified by *a* over $\left[\begin{array}{c} X \end{array}\right]$ under the conditions specified by *b*; *a* and *b* are given in the top row. Unless otherwise stated, the temperature is 1700 ${\;}^{\circ }$C and the pressure is 100 bar.

Species	Temperature (1300 ${\;}^{\circ }$$C_{a}$/1700 ${\;}^{\circ }$$C_{b}$)	Pressure (10 ${\mathrm{bar}}_{a}$/100 ${\mathrm{bar}}_{b}$)
$\left[\begin{array}{c} HCN_{a} \end{array}/\begin{array}{c} HCN_{b} \end{array}\right]$	$10^{-3}$	$10^{-1}$
$\left[\begin{array}{c} HC_{3}N_{a} \end{array}/{\begin{array}{c} HC_{3}N \end{array}}_{b}\right]$	$10^{-5}$	$10^{-3}$
$\left[\begin{array}{c} HNC_{a} \end{array}/\begin{array}{c} HNC_{b} \end{array}\right]$	$10^{-3}$	$10^{-1}$

We find that in all cases, nitrile concentrations change linearly when varying the abundance of N. The results when varying N are therefore not plotted.
